# Prediction of recurrence in early stage non-small cell lung cancer using computer extracted nuclear features from digital H&E images

**DOI:** 10.1038/s41598-017-13773-7

**Published:** 2017-10-19

**Authors:** Xiangxue Wang, Andrew Janowczyk, Yu Zhou, Rajat Thawani, Pingfu Fu, Kurt Schalper, Vamsidhar Velcheti, Anant Madabhushi

**Affiliations:** 1 Case Western Reserve University, 10900 Euclid Ave, Cleveland, OH USA; 2 Cleveland Clinic Foundation, 16761 Southpark Center, Cleveland, 44136 OH USA; 3 Yale University School of Medicine, 333 Cedar St, New Haven, 06510 CT USA

## Abstract

Identification of patients with early stage non-small cell lung cancer (NSCLC) with high risk of recurrence could help identify patients who would receive additional benefit from adjuvant therapy. In this work, we present a computational histomorphometric image classifier using nuclear orientation, texture, shape, and tumor architecture to predict disease recurrence in early stage NSCLC from digitized H&E tissue microarray (TMA) slides. Using a retrospective cohort of early stage NSCLC patients (Cohort #1, n = 70), we constructed a supervised classification model involving the most predictive features associated with disease recurrence. This model was then validated on two independent sets of early stage NSCLC patients, Cohort #2 (n = 119) and Cohort #3 (n = 116). The model yielded an accuracy of 81% for prediction of recurrence in the training Cohort #1, 82% and 75% in the validation Cohorts #2 and #3 respectively. A multivariable Cox proportional hazard model of Cohort #2, incorporating gender and traditional prognostic variables such as nodal status and stage indicated that the computer extracted histomorphometric score was an independent prognostic factor (hazard ratio = 20.81, 95% CI: 6.42–67.52, P < 0.001).

## Introduction

Lung cancer is the most common cause of cancer related mortality in the world^1^. Non-small cell lung cancer (NSCLC) accounts for approximately 80% of all lung malignancies. Early stage NSCLC (stage I-II) patients are typically treated with complete surgical resection of the tumor. However, even after the entire resection of the tumor, 30–55% of patients will develop disease recurrence within the first 5 years of surgery^2^.

The ability to identify patients with high risk for recurrence following surgical resection can help with surveillance plans and potentially personalize adjuvant therapy for these patients^3,4^. There is, unfortunately, a paucity of validated predictive models and companion diagnostic assays for guiding treatment decisions regarding adjuvant chemotherapy in early stage NSCLC patients^4–6^.

Several clinic-pathological factors are known to be associated with recurrence in early stage NSCLC, such as tumor size (T-stage), nodal involvement (N-Stage), and smoking history^7–9^. Numerous studies have suggested the prognostic importance of nuclear morphometric features from Hematoxylin and Eosin (H&E) stained images in the context of various malignancies^10–17^. In NSCLC, malignant cells tend to have abnormally accelerated cell cycles and often manifest with large hyperchromatic nuclei and tend to grow invasively leading to irregular nuclear shapes. Benign cells which are globally regulated by the genetic code tend to be more circular and have a smaller variance in shape and size^18^. Additionally, the nuclear membrane of malignant cells tends to wrinkle in order to accommodate extra chromosomes (i.e., aneuploidy, or aneusomy) or chromatin content due to altered cell division. These changes could potentially be captured by mathematical measurements of nuclear texture (spatial pixel intensity and variation within nuclear area)^19^. Non-tumor cells lacking genetic and chromosomal aberrations differ from fast-duplicating malignant nuclei and display distinct textural patterns.

With the digitization of tissue slides, there has been recent interest in the role of computer assisted digital analysis of pathology specimens. Some research groups have applied computational imaging approaches to digitized tissue slides of lung cancer to predict outcome. Yu *et al*. found that Zernike shape features of nuclei and cytoplasm could stratify patients based on their risk of post-surgery recurrence in both stage I adenocarcinoma and squamous cell carcinoma^17^. They selected the densest image tiles from the whole slide histopathology image of NSCLC to conduct the image analysis and leveraged the machine-learning tools to classify each tile representation into either recurrence or non-recurrence. The authors first build a histomorphometric classifier using nuclear shape and texture features to distinguish the tumors into adenocarcinomas versus squamous cell carcinomas, the area under the receiver operating characteristic (ROC) curve (AUC) being 0.72. Then for each histologic subtype, nuclear morphometric classifiers were constructed to distinguish short-term and long-term survivors (log-rank test p value = 0.0023 for stage I adenocarcinoma, and log-rank test p value = 0.035 for squamous cell carcinoma)^17^.

While Yu *et al*. showed that nuclear shape and texture were clearly implicated in prediction of recurrence in early stage NSCLC, recent work by Friedl *et al*.^20^ suggests more aggressive tumor cells are prone to coordinate as a group and function as large cellular clusters. This, in turn, suggests that quantitative measurements of nuclear architecture and spatial arrangement might be different between low and high risk for disease recurrence in patients with early stage NSCLC. By considering each nucleus in the image as a vertex of a graph and connecting the graph vertices with edges, one can construct different spatial maps (e.g., Voronoi, Delaunay, Minimum Spanning Tree) of nuclear arrangement. Quantitative measurements of nuclear arrangement such as inter-vertex distance or nuclear density can then be mined from these graphical representations. Doyle *et al*.^21,22^ have shown that such representations of nuclear architecture are useful in predicting breast cancer grade. These have also been used for predicting disease progression in p16+ oropharyngeal cancers^10^ and biochemical recurrence in prostate cancer^11^.

In this work, we employ histomorphometric analysis to extract quantitative measurements of nuclear architecture, texture, and shape of the tumor from digitized H&E tissue microarray (TMA) slides and then identify the association of these features with recurrence in early stage NSCLC. Specifically, we employ feature selection to identify the most predictive of the extracted features for identifying patients at high risk for recurrence using a learning cohort of early stage NSCLC patients (Cohort #1, n = 70). The most predictive features associated with disease recurrence are then used in conjunction with a machine learning classifier to build a predictive model for predicting recurrence. The machine learning model was then independently validated in a separate cohort of early stage NSCLC patients (Cohort #2, n = 119); histologic subtype (i.e. squamous or adenocarcinoma) was analyzed independently. Another validation set Cohort #3 (n = 116) of early stage NSCLC patients with two TMA spots for each patients was used to test the robustness of the model to tumor samples obtained from different locations within the resected specimen. A Cox proportional hazards prognostic model^23^ was employed in conjunction with the most predictive features identified on the training set to generate continuous risk scores of recurrence, to enable more continuous and granular categorization of patients into hazard groups.

## Materials and Methods

### Patients and Tissue Microarrays

All experimental protocols in the study were approved by the University Hospitals Cleveland Medical Center (UHCMC) IRB (IRB# NHR-15–55) and were not classified as “human subject research” according to Federal Regulations and were considered HIPAA Exempt. This study included H&E stained sections from three independent retrospective cohorts of NSCLC patients in the form of tissue microarrays (TMA). The deidentified tissue samples were obtained under an existing IRB-approved protocol at the Cleveland Clinic (IRB# 14–562) with Dr. Vamsidhar Velcheti as the PI which allows the use of radiographic images, histologic slides, and archival tissue available at the Clinic since 01/01/1990.

**Figure 1:**
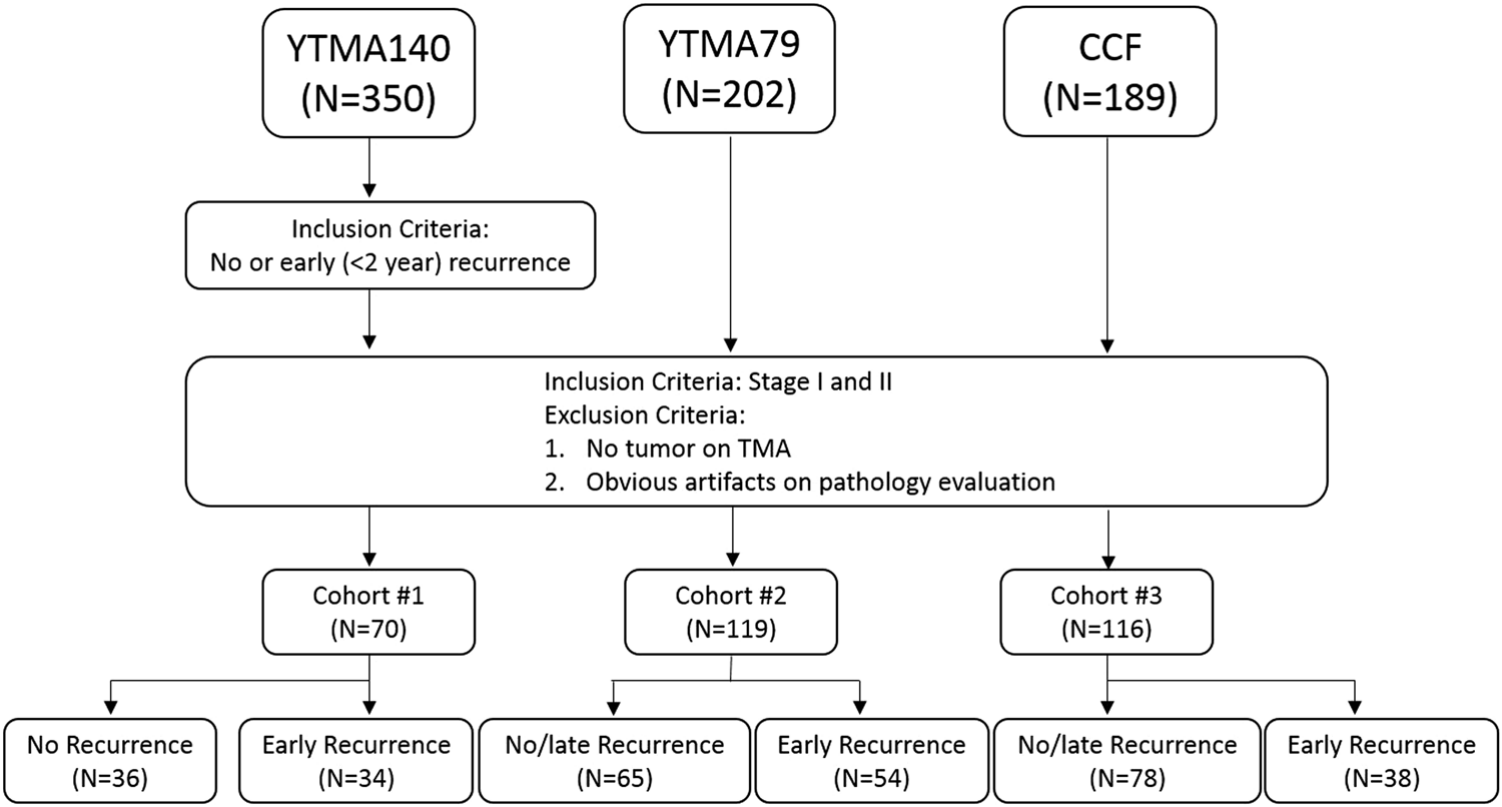
Inclusion and exclusion criteria for patient selection for the training and test sets.

Cohort #1 (total n = 350) was collected in Greece between 1991 and 2001. Cohort #2 (n = 202) was collected at Yale Pathology between 1988 and 2003. Cohort #3 (n = 189) was collected at Cleveland Clinic between 2004 and 2014. In Cohort #3, two tissue punches from different physical locations of the same tumor sample were identified, prepared, and scanned. Additionally, the two tissue punches for each patient were prepared in separate batches (batch #1 and batch #2) at the time of the tissue acquisition. All the patients received definitive surgery, either lobectomy or pneumonectomy with lymph node dissection as the primary treatment. Clinical and pathological variables of patients in both cohorts were extracted from clinical records and pathology reports. TMAs were produced by standard procedures via surgical specimens and digitally scanned at 20x, each sample of a single patient was represented by a 1500 pixel × 1500 pixel image. A pathologist, to exclude absence of tumor in the sample, visually inspected each image sample. Only stage I and stage II patients were included in this study. Figure 1 illustrates the criteria for patient selection.

### Nuclear Segmentation

A deep learning approach previously presented in refs^24,25^ was applied to accurately segment individual nuclei in each of the TMA spots, both in the training and validation sets. We adapted popular convolutional neural networks (CNN) to an adaptive architecture resolution, which finds pixels at a low magnification are likely to be nuclei, and solely investigated those pixels at a high magnification, obviating the computational burden of examining all pixels at the high magnification^24^. The result of the nuclear segmentation algorithm was the centroid and boundary for each nucleus identified in each of the TMA spots. A watershed^26^ based segmentation approach was also included as a comparison to the deep learning approach. The second segmentation approach enabled us to comprehensively study the effect of nuclear segmentation on the extracted features and the subsequent prognosis prediction.

### Feature Extraction

Two hundred forty two image features were extracted from only pixels corresponding to the nuclei identified in the TMA spots. The feature categories are described below:

- *Global graph features* (51 descriptors) - Each nuclear centroid was designated as a graph node and then all the nodes in each TMA spot were connected to construct a variety of global nuclear graphs. Depending on the type of connectivity between the nodes, i.e. Voronoi diagram^27^, Delaunay triangulation^28^, or minimum spanning tree^28^, three different global graphs were constructed for each TMA spot. From each global graph, 51 descriptors capturing the topology and spatial relation of nuclei were captured.
- *Local Nuclear cluster graphs* ^10,11^ (26 descriptors) – This version of the nuclear graph involves first identifying clusters of nuclei and then subsequently identify the centroid of the cluster. A cluster is defined by a local aggregation of proximally located nuclei. Cluster centroids are then used to define the nodes of the cluster graph. A similar set of topological and spatial relationship attributes are then mined from each local cluster graph defined on each TMA spot^11^. Unlike the global graph that reflects the micro-level, granular architecture of all individual nuclei in the spot, the local cluster graph appreciates more macro-level, coarser nuclear arrangement.
- *Nuclei shape features* (100 descriptors) – A series of nuclear shape features are extracted from the segmented boundary of each nucleus. These include nuclear area, perimeter, min/max radius and Fourier transform of the nuclear contour^29^.
- *Nuclei orientation entropy* ^14^ (39 descriptors) – In ref.^14^ Lee *et al*. identified the entropy associated with the nuclear directionality in prostate cancer pathology images and showed that higher nuclear disorder in orientations was associated with higher risk of biochemical recurrence following radical prostatectomy. The same approach is employed to measure the entropy and associated disorder of nuclear orientations, the assumption being that recurrent disease will have higher nuclear orientation disorder and associated entropy compared to non-recurrent disease. The directionality of each nucleus was determined by performing principal component analysis on the Cartesian coordinate locations on the set of boundary points of each nucleus. Second order statistics are calculated (e.g., contrast energy, entropy) on the orientation of all nuclei within local clusters. A total of 13 second-order nuclear orientation statistics are, thus, obtained for each nuclear cluster and the mean, median, and standard deviation measurements for each of these statistics aggregated across all the clusters in the TMA spot image.
- *Nuclei texture* (26 descriptors) – Gray level co-occurrence features which capture second order joint intensity statistics are employed to encode the textural heterogeneity of each nucleus. A total of 13-second order texture features are computed and for each feature, the corresponding mean and standard deviation values are measured for each TMA spot.

Details regarding the 242 feature descriptors are provided in supplementary material Table 1.

**Table 1:** Demographic and clinical characteristics of patients in Cohort #1, Cohort #2 and Cohort #3.

	Training cohort (Cohort #1, N = 70)	Validation cohort (Cohort #2, N = 119)	Validation cohort (Cohort #3, N = 116)
Median age	62.4	65.4	66.8
Gender			
Male (%)	84.3	47.9	55.2
Female (%)	15.7	52.1	44.8
Stage			
I (%)	64.3	81.1	66.4
II (%)	35.7	18.9	33.6
T-Stage			
T1 (%)	19.1	51.3	51.7
T2 (%)	80.9	48.7	48.3
N-Stage			
N0 (%)	74.3	53.8	79.3
N1 (%)	25.7	46.2	20.7
Non-recurrence (%)	51.4	54.6	67.2
Recurrence (%)	48.6	45.4	32.8

To alleviate the issue of batch effects [24], a term that refers to variances shared by a set of specimens undergoing similar preparation steps (e.g., staining and digitization^30^), color normalization was applied to all the images using the non-linear spline mapping approach described in ref.^31^.

### Feature Selection

Feature selection was employed to identify a subset of features that were most discriminating of patients who had early versus no disease recurrence from within the larger set of 242 total features. A variant of the Minimum redundancy maximum relevance (mRMR)^32^, a feature selection approach that uses mutual information as a similarity measure, was employed to find a subset of the most discriminative features. The process of feature selection was only applied to the cases within the training set (Cohort #1). mRMR aims to identify a combination of features which together could maximize the joint dependency for distinguishing binary classes (in this case recurrence vs. non-recurrence) while minimizing the redundancy within the feature combination. However, our implementation of mRMR involved using it to identify the top 3 most highly ranked features within each feature category. Across the 5 feature categories this yielded a total of 15 features. From within this set of 15 features, the best combination (7 features out of 15) was finally determined via quadratic discriminant analysis (QDA). The QDA scheme was optimized across 100 iterations of 3-fold cross-validation on the training set.

### Classifier construction

The classifiers implemented in our study consisted of quadratic discriminant analysis (QDA), linear discriminant analysis (LDA), and support vector machine (SVM). We fed the identical subset of features identified as being most predictive of recurrence and non-recurrence in our feature selection step to construct the three different classifiers. 70 cases from Cohort #1 were used to train and lock down the 3 different classifiers.

### Statistical Analysis

**Figure 2:**
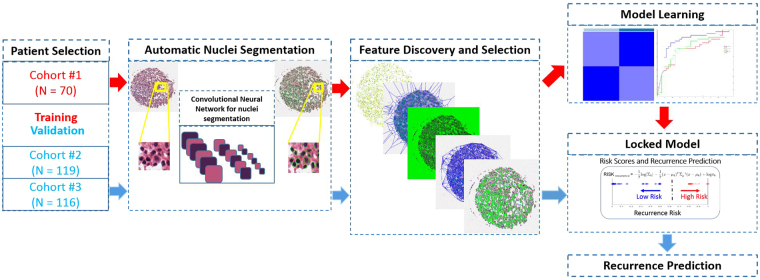
Flowchart illustrating the procedure for training and validating the quantitative histomorphometric classifier for distinguishing early versus no/late recurrence in early stage lung cancer.

The overall workflow for the construction and subsequent validation of the histomorphometric image based classifiers for early stage lung cancer is shown in Fig. 2. To validate the prediction models constructed during the training phase, the following series of steps were employed. First, each digitized TMA image in the test set is fed to the deep learning network for segmentation of the individual nuclei. Second, image features identified as most predictive of early versus no or late recurrence during the learning phase by mRMR are extracted.

Three different classifiers (QDA, LDA, SVM) were trained on the learning set and then subsequently evaluated in their ability to predict risk of recurrence on the test set. Area under the Receiver operator characteristic (ROC) curve (AUC) for each classifier was evaluated in distinguishing early versus no or late disease recurrence on the training and test sets. Additionally, the Kaplan-Meier method^33^ was used to correlate the recurrence-free survival (RFS) which was measured from the date of diagnosis to the date of death or the date of disease recurrence whichever occurred first and censored at the date of last seen for those still alive without recurrence with the best classification results. Difference of RFS among predicted categories was examined using a log-rank test. Associations between the true recurrence labels and major clinical categorical variables were found by Fisher exact test^34^. A Multivariable Cox proportional regression model^35^ was employed to test the independent predicting capability of the classifier on recurrence-free survival after taking major clinical parameters into account. A second Cox proportional hazards prognostic model^23^ was employed in conjunction with the image features identified on the training set. This model was used to generate continuous risk scores for patients in the validation set. The risk score is a weighted sum of the image features, where the weights are the regression coefficients. These risk scores are critical as they can enable categorization of patients into more granular hazard groups. Additionally, similar to Yu *et al*. in ref.^17^, we also developed and evaluated histologic subtype classifiers (i.e. adenocarcinomas and squamous cell carcinomas) to assess whether the features identified as prognostic were different between the two NSCLC subtypes. All tests were 2-sided and a 0.05 significance level was set for this study. Hazard ratio (HR) and its 95% confidence interval were reported. All statistical analyses were performed using MatLab.

## Results

The 70 patients in Cohort #1 were employed for feature discovery and classifier training during the learning phase. The 119 patients in Cohort #2 were used for independent validation. The 116 patients in Cohort #3, two tissue punches from different physical locations of the same tumor sample, were used to quantitatively assess the ability of our approach to deal with intra-tumoral heterogeneity. The two major NSCLC subtypes, adenocarcinoma and squamous cell carcinoma, comprised 17 and 44 in the learning Cohort #1, 51 and 21 cases in the validation Cohort #2, 54 and 20 cases in validation Cohort #3, respectively. Baseline characteristics of the 3 cohorts are summarized in Table 1. The median follow-up for patients was 40.91 months, 45.33 months, and 70.94 months for Cohort #1, Cohort #2 and Cohort #3, respectively. By the end of the study/follow-up, 34 out of 70 patients (48.6%) in Cohort #1, 54 of 119 (45.4%) in Cohort #2, and 38 out of 116 (32.8%) in Cohort #3 had developed recurrence. Correlation between these clinic-pathological factors and patient outcome was calculated by the Fisher exact test and the results illustrated in Table 2.

**Table 2:** Statistical significance test by Fisher’s exact test between gender, major pathological characteristics and disease outcome of patients in Cohort #2 and Cohort #3 set.

Characteristic	Cohort #2 = 119, N (%)	No-Recurrence = 65, N (%)	Recurrence = 54, N (%)	P	Cohort #3 = 116, N (%)	No-Recurrence = 78, N (%)	Recurrence = 38, N (%)	P
Gender								
Male	57(47.9)	30 (46.2)	27(50.0)	0.7151	64	44	20	0.8425
Female	62(52.1)	35(53.8)	27(50.0)		52	34	18	
T Pathological								
T1	61(51.3)	35(53.8)	26(48.1)	0.5834	60	43	17	0.3267
T2	58(48.7)	30(46.2)	28(51.9)		56	35	21	
N Pathological								
N0	64(53.8)	36(55.4)	28(51.9)	0.7159	92	63	29	0.6287
N1	55(46.2)	29(44.6)	26 (48.1)		24	15	9	

Tow-sided P < 0.05 was considered as statistically significant.

**Figure 3:**
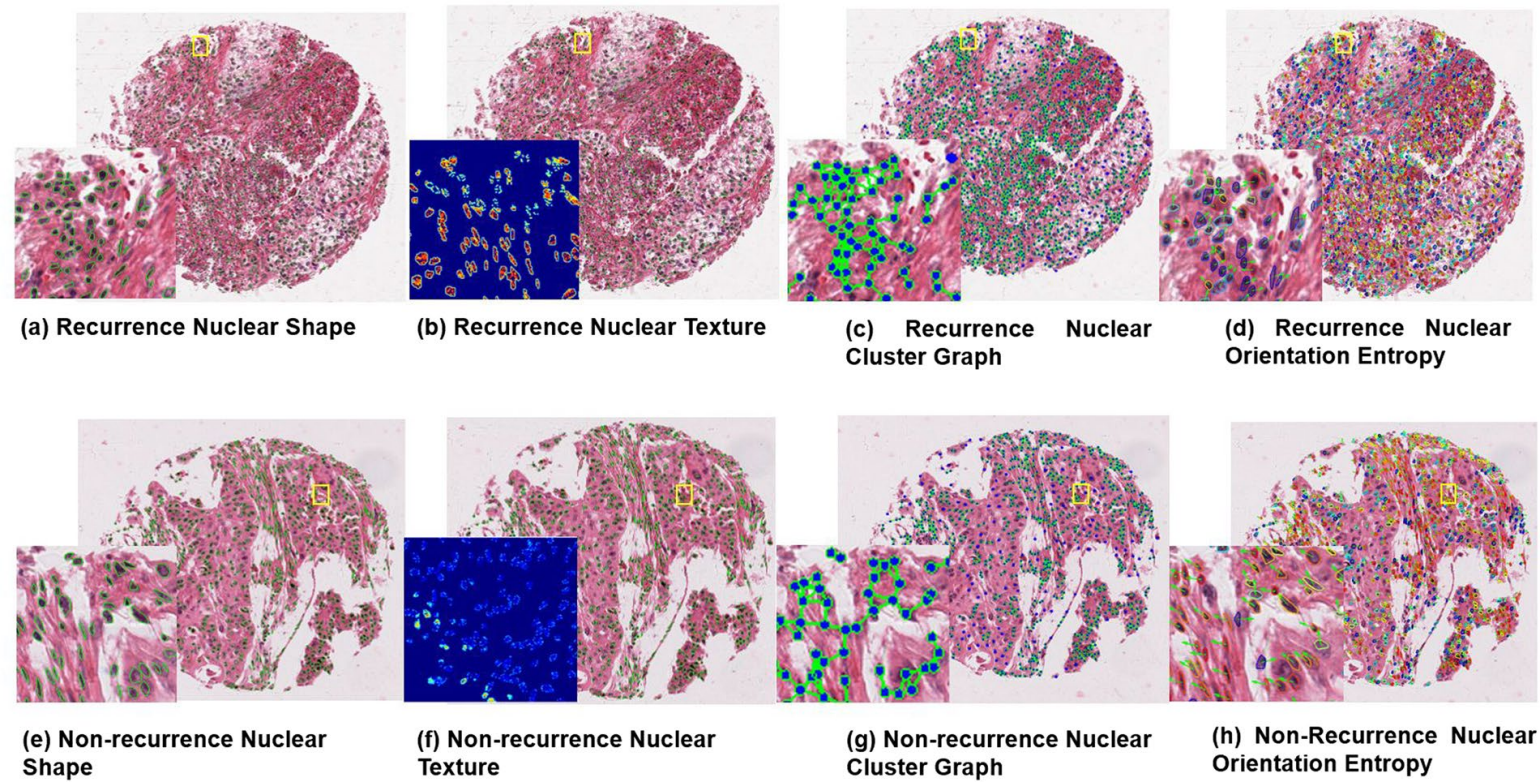
Representative TMA tissue spots of recurrent (top row) and non-recurrent (bottom row) NSCLC with corresponding feature maps: Recurrence TMA with (**a**,**e**) nuclear shape feature, (**b**,**f**) texture feature map (Haralick standard deviation intensity correlation), (**c**,**g**) nuclear cluster graph feature map, and (**d**,**h**) nuclear orientation.

The seven most predictive image features identified on the learning set were nuclear graph (2), shape (2), and texture (3) (Table 3 and Fig. 3). The top graph features included ratio of minimum and maximum area of polygons within the nuclear Voronoi graph and average number of nearest neighbors within a 40-pixel radius. Mean of Fourier shape descriptor 4 and min/max ratio of Fourier shape descriptor 8 were identified as the most discriminating shape features. The Haralick descriptors included standard deviation of contrast variance, contrast energy, and contrast inverse moment. The distribution of values for these seven features is included in supplementary material Fig. 1.

**Table 3:** A subset of 7 features selected from entire feature set.

Feature category	Description
Graph	Voronoi: Area Ratio Minimum / Maximum
Graph	Arch: Average Nearest Neighbors in a 40 Pixel Radius
Shape	Min/max ratio of Fourier Descriptor 8
Shape	Mean of Fourier Descriptor 4
Texture	Haralick standard deviation intensity contrast variance
Texture	Haralick standard deviation intensity contrast energy
Texture	Haralick standard deviation intensity contrast inverse moment

**Figure 4:**
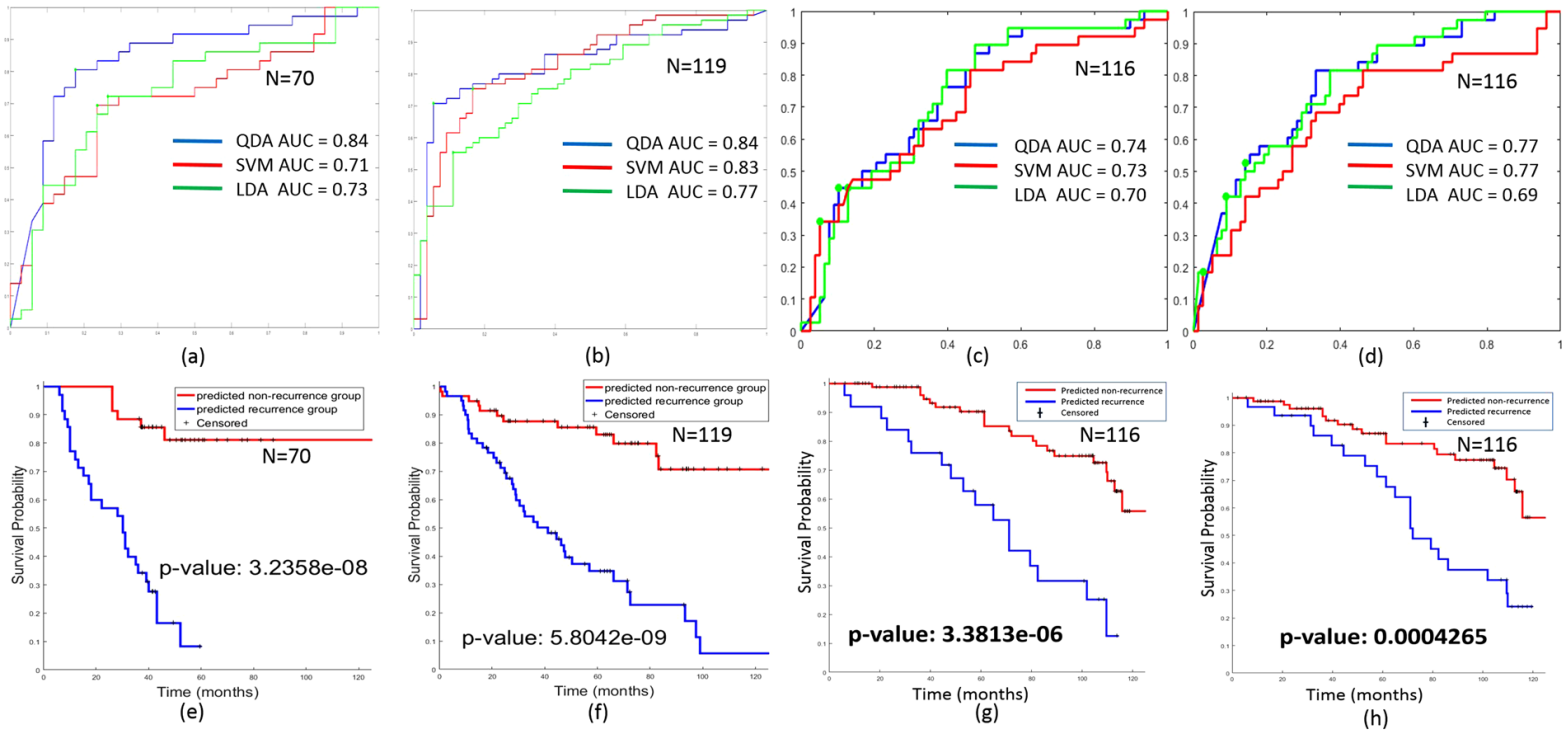
ROC analysis of classifier predicting recurrence on (**a**) training set Cohort #1, (**b**) independent validation set Cohort #2, (**c**) independent validation set Cohort #3 batch #1 and (**d**) independent validation set Cohort #3 batch #2 show consistent predicting ability among different classifiers and among different tumor section.Kaplan-Meier survival analysis for (**e**) training set Cohort #1 and (**f**) validation set Cohort #2 (**g**,**h**) batch #1 and batch #2 from Cohort #3 show good visual separation and log-rank test indicates the two groups were statistically different (p-value ≪ 0.05).

On both the training and validation sets, the QDA classifier was found to be the most predictive in terms of AUC (Fig. 4a~d). The QDA classifier yielded an AUC = 0.84, 0.74 and 0.77 and an accuracy of 82%, 75% and 75% in distinguishing between recurrent and non-recurrent early stage lung cancers in Cohort #2, Cohort #3 batch #1 and batch #2. Among the 54 recurrent patients within the validation set Cohort #2, our QDA classifier successfully predicted 51 as recurrence; with a recall of 94.4% (only 3 recurrent patients were missed). Overall, the model predicted 70 cases as recurrence with 51 true positive cases, resulting in a positive predictive value (PPV) of 72.9% on Cohort #2. The details of model prediction are presented in the form of a confusion matrix (Table 4).

**Table 4:** Classification results with real patient 5-year outcomes of validation set Cohort #2 and Cohort #3.

	5-year recurrence	No 5-year recurrence
Classifier-recurrence, Cohort #2	51	19
Classifier-non-recurrence, Cohort #2	3	46
Classifier-recurrence, Cohort #3 batch #1	17	8
Classifier-non-recurrence, Cohort #3 batch #1	21	70
Classifier-recurrence, Cohort #3 batch #2	20	11
Classifier-non-recurrence, Cohort #3 batch #2	18	67

Kaplan-Meier survival curves of the QDA model were plotted for the training (Fig. 4e, log- rank p = 3.2e-8) and validation sets (Fig. 4f,g, log-rank p ≪ 0.05). In both the training and validation cohorts, patients predicted as being recurrent by the model had statistically significantly worse overall survival. A multivariable Cox proportional hazard model was also employed in this study to validate the independence of the 7-feature model employed in the classifier after controlling for the effects of other prognostic co-variables (see Table 5). The estimated hazard of having disease recurrence in classifier predicted recurrence group is 20.8 times of that in the predicted non-recurrence group for Cohort #2; that is a 20-fold increase of the recurrence risk after adjustment for the other variables. Moreover, the 95% confidence interval of hazard ratio ranged from 6.41 to 67.5, with a p-value less than 0.0001.

**Table 5:** Multivariable Cox proportional hazard model controlling for major pathological variables on validation set Cohort #2 and Cohort #3, batch #1 and batch #2.

	Characteristic	hazard ratio (95% CI)	P - value
Cohort #2	Gender (Male Vs. Female)	1.3046 (0.753, 2.26)	0.343
	T Pathological (T1 vs. T2)	1.0737 (0.433, 2.664)	0.878
	N Pathological (N0 vs. N1)	1.1961 (0.477, 3)	0.703
	Classified (Non-Recurrence vs. Recurrence)	20.812 (6.415, 67.52)	<0.0001
Cohort #3, batch #1	Gender (Male Vs. Female)	0.9274 (0.449, 1.916)	0.839
	T Pathological (T1 vs. T2)	1.7725 (0.905, 3.473)	0.095
	N Pathological (N0 vs. N1)	1.8686 (0.811, 4.307)	0.142
	Classified (Non-Recurrence vs. Recurrence)	4.6532 (2.294, 9.44)	<0.0001
Cohort #3, batch #2	Gender (Male Vs. Female)	0.7885 (0.385, 1.614)	0.516
	T Pathological (T1 vs. T2)	1.4941 (0.768, 2.909)	0.237
	N Pathological (N0 vs. N1)	1.8984 (0.842, 4.28)	0.122
	Classified (Non-Recurrence vs. Recurrence)	2.9239 (1.476, 5.791)	0.002

P-values in bold are statistically significant.

The monolithic prediction model trained on both ADCs and SCCs performed comparably to the models individually trained on ADCs and on SCCs (see supplementary material, Fig. 2). In prediction of recurrence in ADCs subtype, the model trained on combined subtypes achieved overall better prediction (AUC = 0.86, 0.90 and 0.75 via QDA, SVM and LDA) on validation Cohort #2 compared to the model trained only on ADCs (AUC = 0.73, 0.75 and 0.76 via QDA, SVM and LDA). Additionally, improved prediction performance for SCCs was also observed when the model was trained using a combination of ADCs and SCCs (see supplementary material, Fig. 2b,d).

Training the Cox regression model with the same seven features used to develop the classification model gave us an opportunity to calculate the risk score^23^ for recurrence for each patient. Based on the risk score obtained for the 119 patients from the validation Cohort #2 via the Cox regression model, we further stratified validation Cohort #2 into 2 and 3 sub-sets respectively. This patient grouping into 2 and 3 different sub-groups was done based on the median and tertiles of the risk scores (see Supplementary Figure 3). The median and tertiles of risk scores were determined on the training and validation set separately (median = −2.5460, tertiles = −2.8509; −2.1795 for training set Cohort #1 and median = −1.3787, tertiles = −2.0100; −0.8883 for validation set Cohort #2). The same risk scores and grouping experiments were also conducted on validation Cohort #3 batch #1 and batch #2 (median = −3.3249, tertiles = −3.7004; −2.8515 for validation Cohort #3 batch #1 and median = −3.4624, tertiles = −3.7960; −2.9218 for validation Cohort #3 batch #2). We observed that the patients in the validation sets (Cohorts #2, #3) were stratified based off time to recurrence (early, intermediate and delayed) in a way that was statistically significantly different between the 3 risk (early, intermediate and delayed) groups, not just for the 2 risk (early and late) groups (see Supplementary Figure 3).

In Cohort #3, the prediction model yielded similar classification results on batch #1 and batch #2: 0.74 vs 0.77 respectively via a QDA classifier (see Supplementary Figure 4a). The differences were not statistically significantly different (p = 0.8551, by McNemar’s test) between batches #1 and 2, suggesting that the prediction model was robust to location of tumor sample from the resected specimen. Additionally, the Kaplan-Meier survivial analysis for both batch #1 and batch #2 showed significant separation of the different survival groups (p-value = 3.3e-06 vs. 0.00043 for batch #1 and batch #2, see Fig. 4b and c in the Supplementary section).

Nuclei were segmented by the watershed approach described in ref.^26^ on Cohorts #1 and #2. All other parameters in the downstream feature extraction and training schemes remained unchanged. The classifier trained by the watershed algorithm was found to be comparable to the deep learning based approach (AUC = 0.82 vs 0.84, respectively, p-value = 0.2478 by McNemar’s test, see Figure 5 in the Supplementary section). Difference in the total number of nuclei identified by the two segmentation approaches was found to be small (see Figure 6 in Supplementary section).

## Discussion

In this study, we developed a computer assisted histomorphometric classifier to predict risk of recurrence in early stage NSCLC based off digital TMA spots of surgically excised tissue specimens. The approach involved computerized extraction of nuclear shape, texture, and architecture features and then identifying the combination of features that were most predictive of recurrence in early stage NSCLC on a training set. The features were independently validated for their ability to distinguish recurrence in patients with early stage NSCLC patients, in conjunction with a machine learning classifier. Our results showed that the combination of nuclear shape, texture, and architectural features were predictive of recurrence in early stage NSCLC, independent of clinical parameters such as gender, cancer stages, and histologic subtype. Moreover, our results appear to suggest that the image classifier is able to predict disease outcome independent of the spatial location of where in the tumor block the tissue punch came from and independent of different nuclei segmentation methods as well. Continuous risk scores computed via the Cox proportional hazard model allowed for assigning individual patients into more specific risk groups based on computed individual hazard ratios.

Nuclear and quantitative histomorphometric analysis is gaining a great deal of interest in the context of risk stratification of a number of solid tumors^14,36^. Additionally there is increasing evidence of tumor behavior being a consequence of the coordinated activity and architecture of cell groups rather than individual cells^37,38^. Tumor cells, more so than healthy cells, tend to aggregate into clusters. This cluster behavior allows cancer cells to potentially expand, progress, and metastasize^37,38^. As a result, it seems plausible that features that capture and characterize this clustering property might enable differentiation of the high versus lower risk tumors. Consequently, in this work we decided to specifically focus on the role of nuclear graph features to model the arrangement of clusters of cancer cells, and, hence, predict tumor behavior in early stage NSCLC. The two most predictive nuclear graph features were determined to be (1) ratio of minimum and maximum area of polygons within the nuclear Voronoi diagram, and (2) average number of nearest neighbors within a 40 pixel radius of each node within the nuclei graph; features that reflect the variance in spatial proximity of cancer nuclei. These features might reflect the fact that lower risk tumors (i.e. no or delayed recurrence) have a more coherent nuclear architecture and organization when compared to higher risk, early recurrent tumors^12,14^. In addition to nuclear architecture, nuclear shape and nuclear texture features, previously implicated in recurrence of NSCLC^17^, were also validated to be prognostic.

Three popular classifiers (QDA, LDA, and SVM with polynomial kernel) were built upon the nuclear morphologic features identified as most predictive on the training set. While the QDA classifier was found to be marginally superior compared to the SVM and LDA classifiers, all 3 classifiers were found to (1) be prognostic on the independent test set resulting in statistically significant separation between the recurrence and non-recurrence groups in Kaplan-Meier cumulative hazard analysis, and (2) superior compared to other current clinical or pathological parameters^8^. A classifier based off tumor stage (T1 versus T2) and nodal status (N0 versus N1) only resulted in predictive accuracy of 55.8% on the test set, significantly lower compared to the machine based classifiers (72%–82%). More importantly, the consistency in performance of the three different classifiers reaffirmed the prognostic accuracy of the nuclear morphologic features identified.

Multivariable cox proportional hazard model in conjunction with the 7 nuclear morphologic features suggested a significant increase in the hazard ratio for patients identified with early recurrent disease (p < 0.0001). The features were found to be prognostic of recurrence, even after controlling for the effect of other clinical and pathological variables. No other clinic-pathological parameters were found to be prognostic in the multivariable analysis. Additionally, these results of the histomorphometric classifier were not found to be significantly different when controlling for the histologic subtype of the tumor (i.e. adenocarcinoma vs squamous tumor).

The closest related work to our study is that of Yu *et al*.^17^ and David H *et al*.^8^, both studies having explored the role of histomorphometric image analysis of tissue specimens for predicting disease outcomes for NSCLC patients. Yu *et al*. reported that the Zernike shape features of nuclei were predictive of recurrence in NSCLC adenocarcinoma and stage I squamous cell carcinoma. Our approach differs from that of Yu *et al*.^17^ and David H *et al*.^8^ in that apart from being fully automated for nuclear detection, segmentation and feature extraction, our approach also invoked additional features apart from the nuclear shape and texture features. Specifically, we employed four additional feature categories focused on quantitatively characterizing nuclear architecture and directionality; these features identified on our learning set were highly predictive of disease recurrence. Finally, our results on the independent validation suggest significantly higher accuracy in predicting disease recurrence (see Figure 7 in the Supplementary Section), both in terms of classifier accuracy and Kaplan-Meir curve analysis when compared to the results reported in refs^8,17^.

Our study did have its limitations. First, the analysis was performed using TMAs, which represent only a relatively small portion of the tumor. However, it is noteworthy that the predictive power of the features is present even in small TMA spots extracted from the larger surgical resections. Moreover, these features were able to accurately distinguish early and late/no recurrence patients after being surgically treated for early stage NSCLC. While, we found that the histologic subtype of the tumor did not appear to have a significant bearing on the classifier accuracy, we did not have access to the molecular subtypes of the tumors, PD-L1 expression or TILs abundance. Hence, we were unable to relate whether the better and worse prognostic cases were related to specific oncogenic mutations (e.g. EGFR, KRAS, ALK, and ROS1) or immune features. Future work will test our classifier in conventional whole slide images to capture and relate intra-tumoral heterogeneity to disease outcomes, and attempt to correlate the prognostic histomorphometric features with the underlying tumor biology.

## Concluding Remarks

In summary, computer-extracted nuclear feature analyses of digitized slides of NSCLC biopsy specimen may enable objective and reproducible prediction of recurrence and disease outcome in patients with early stage NSCLC. With additional prospective/multi-site validation, this prognostic model could potentially serve as a predictive decision support tool for deciding the use of adjuvant treatment in early stage lung cancer.

## Electronic supplementary material

Supplementary Material
