# Supplementary material for: Prediction of recurrence in early stage non-small cell lung cancer using computer extracted nuclear features from digital H&E images
Source: Sci Rep. 2017 Oct 19;7:13543. doi: 10.1038/s41598-017-13773-7 (PMC5648794; doi:10.1038/s41598-017-13773-7)
Supplement: Supplementary file 1 — Supplementary Material [file 41598_2017_13773_MOESM1_ESM.pdf]

Prediction of recurrence in early stage non-small cell lung cancer using computer extracted nuclear features from digital H&E images

Xiangxue Wang[1], Andrew Janowczyk[1], Yu Zhou[1], Rajat Thawani[1], Pingfu Fu[1], Kurt Schalper[3], Vamsidhar Velcheti[2], and Anant Madabhushi[1],*

1Case Western Reserve University, OH, USA

2Cleveland Clinic Foundation, OH, USA

3 Yale University School of Medicine, CT, USA

*anant.madabhushi@case.edu

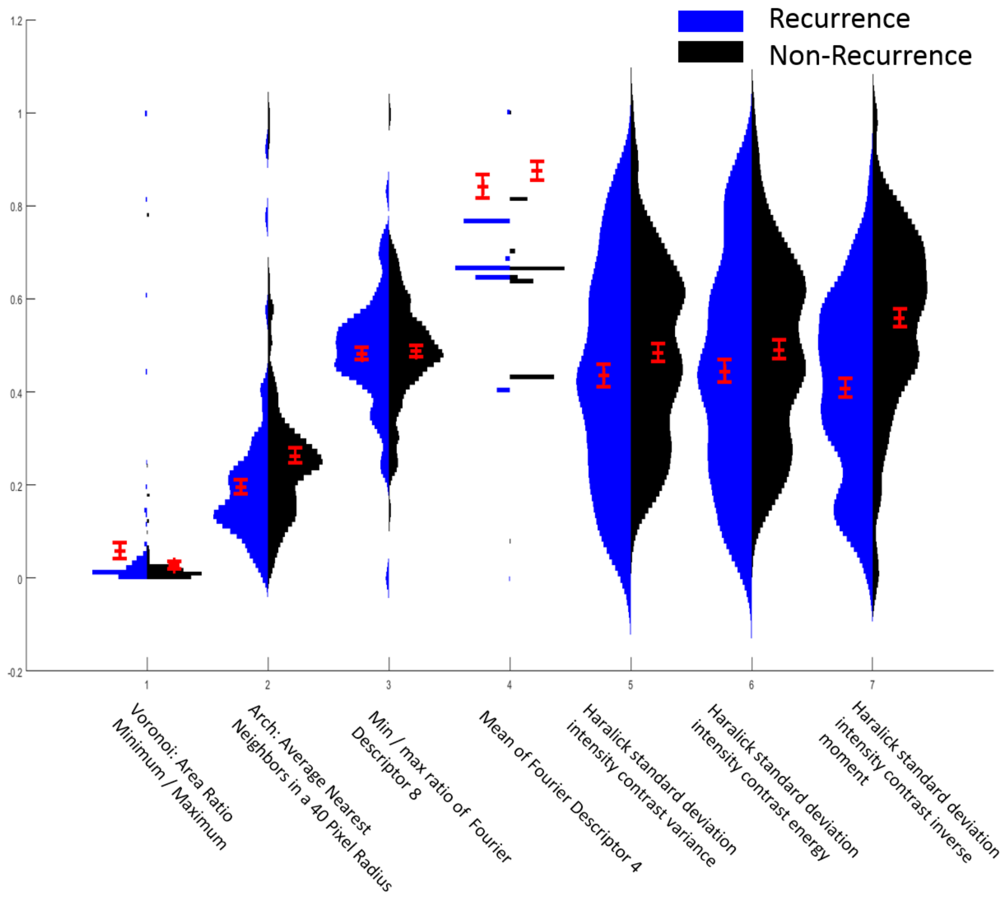

Figure 1. Grouped violin plot of top 7 features on entire TMA spots (YTMA140, n=70 and YTMA79, n=119). The mean with +/- standard error of the mean are indicated via red bars. The distribution of each feature for recurrence and non-recurrence patients shows different trends with different mean values, reflecting the predictive ability of our selected image features.

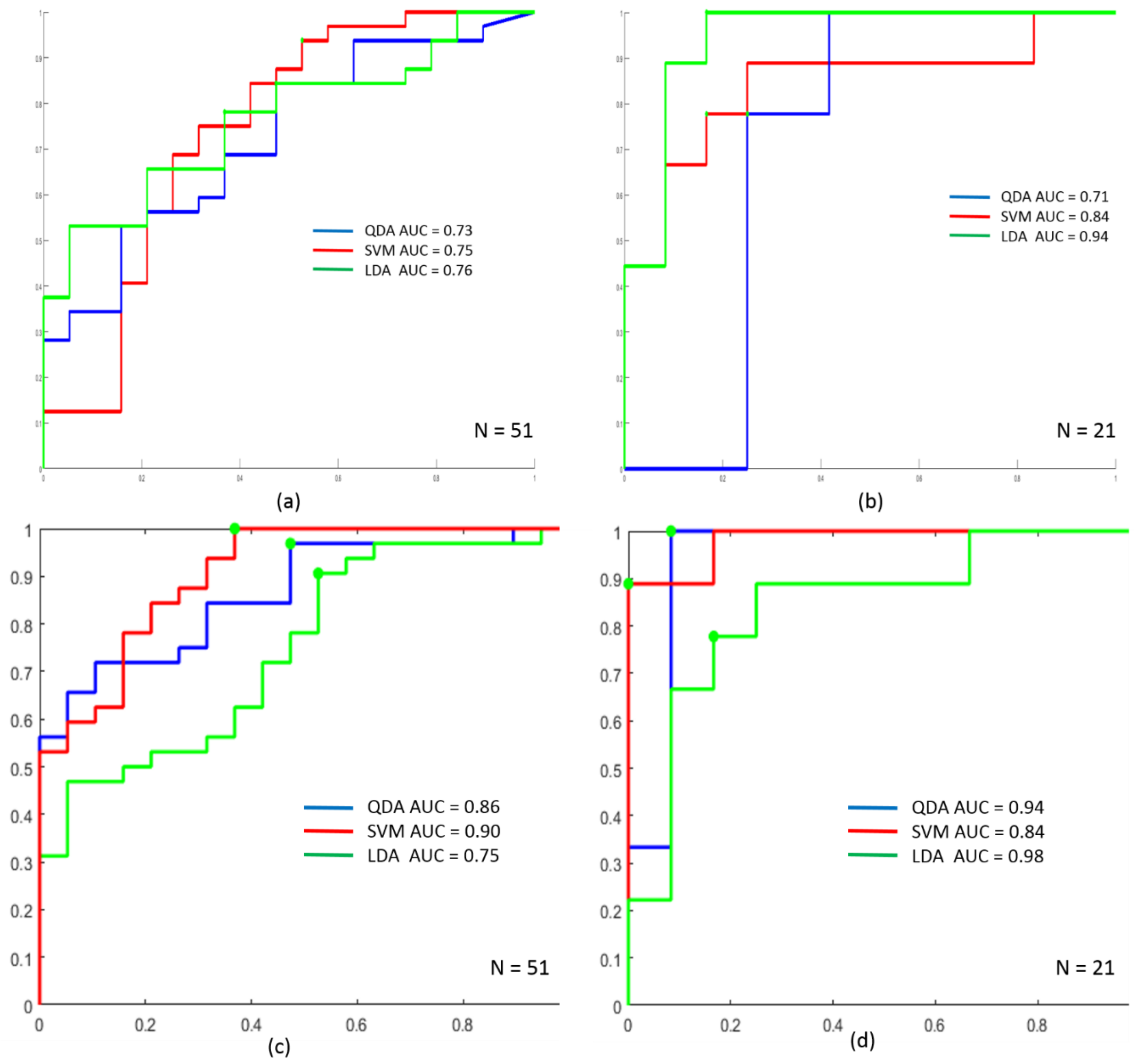

Figure 2. (a) ROC analysis of classifiers on independent validation set cohort #2 of lung adenocarcinoma (ADC), trained only on lung ADC for cohort #1; (b) ROC analysis of classifiers on independent validation set cohort #2 of lung squamous cell carcinoma (SCC), trained only on lung SCC for cohort #1; (c) ROC analysis of classifiers on independent validation set cohort #2 of lung ADC, trained on both lung ADC and SCC for cohort #1; (d) ROC analysis of classifiers on independent validation set cohort #2 of lung SCC, trained on both lung ADC and SCC for cohort #1. Classifiers are predictive for both sub-types with even better performance when trained on the combined set of ADC and SCC patients.

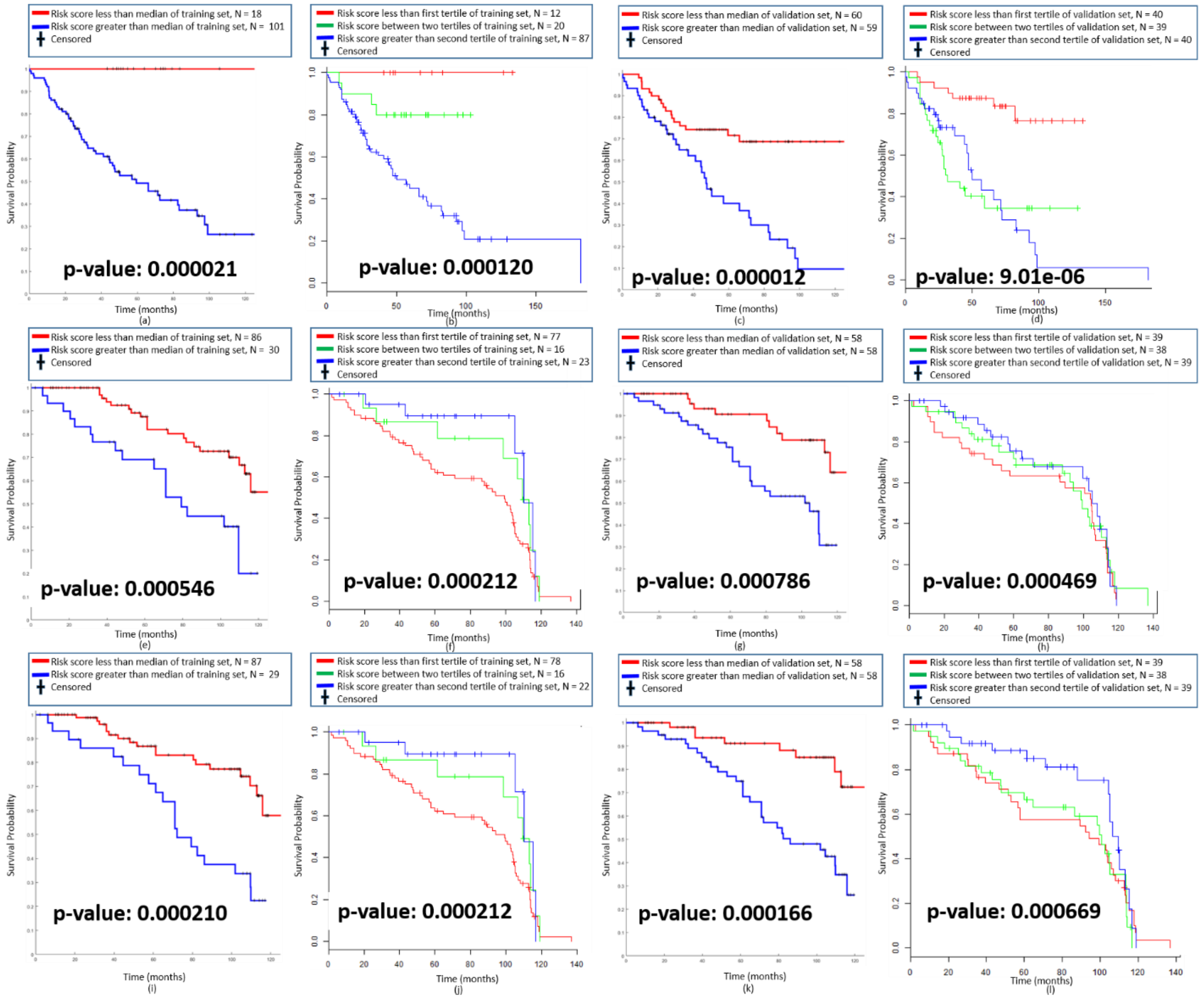

Figure 3. Kaplan Meier curve analysis on validation set (Cohort #2) by categorization of risk scores via cox regression model into (a) 2 and (b) 3 risk groups based off the median (-2.5460) and tertiles (-2.8509 and -2.1795) of risk scores determined on the training set separately and (c) 2 and (d) 3 risk groups based off the median (-1.3787) and tertiles (-2.0100 and -0.8883) of risk scores determined on the validation directly; on validation set (Cohort #3, batch #1) by categorization of risk scores via cox regression model into (e) 2 and (f) 3 risk groups based off the median (-2.5460) and tertiles (-2.8509 and -2.1795) of risk scores determined on the training set separately and (g) 2 and (h) 3 risk groups based off the median (-3.3249) and tertiles (-3.7004 and -2.8515) of risk scores determined on the validation directly; on validation set (Cohort #3, batch #2) by categorization of risk scores via cox regression model into (i) 2 and (j) 3 risk groups based off the median (-2.5460) and tertiles (-2.8509 and -2.1795) of risk scores determined on the training set separately and (k) 2 and (l) 3 risk groups based off the median (-3.4624) and tertiles (-3.7960 and -2.9218) of risk scores determined on the validation directly. Grouping based off median and tertiles from either training or validation cohort yielded statistically different survival groups, while cutoff points directly from the validation cohort yielded a lower p-value overall.

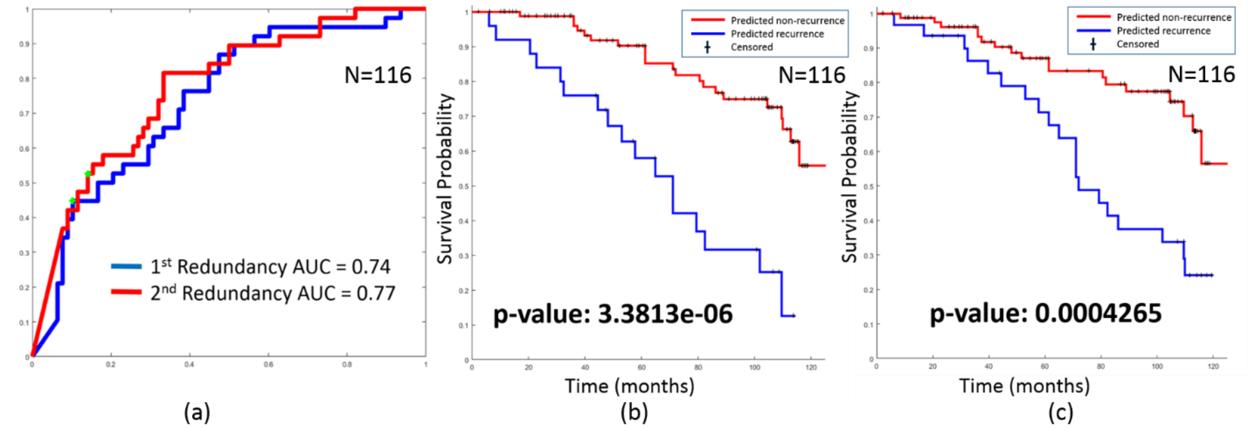

Figure 4. ROC and Kaplan Meier analysis of classifier predicting on independent validation Cohort #3 (n=116): (a) AUC for batch #1 and batch #2 containing different samples from the same 116 patients show similar results (p = 0.8551, by McNemar's test); Kaplan-Meier analysis for (b) patients in batch #1 and (c) patients in batch #2. Again the survival curve has similar trends and separation. In both cases, the log-rank test indicates a statistically significant difference between predicted recurrence and non-recurrence groups for both batches (p-value << 0.05).

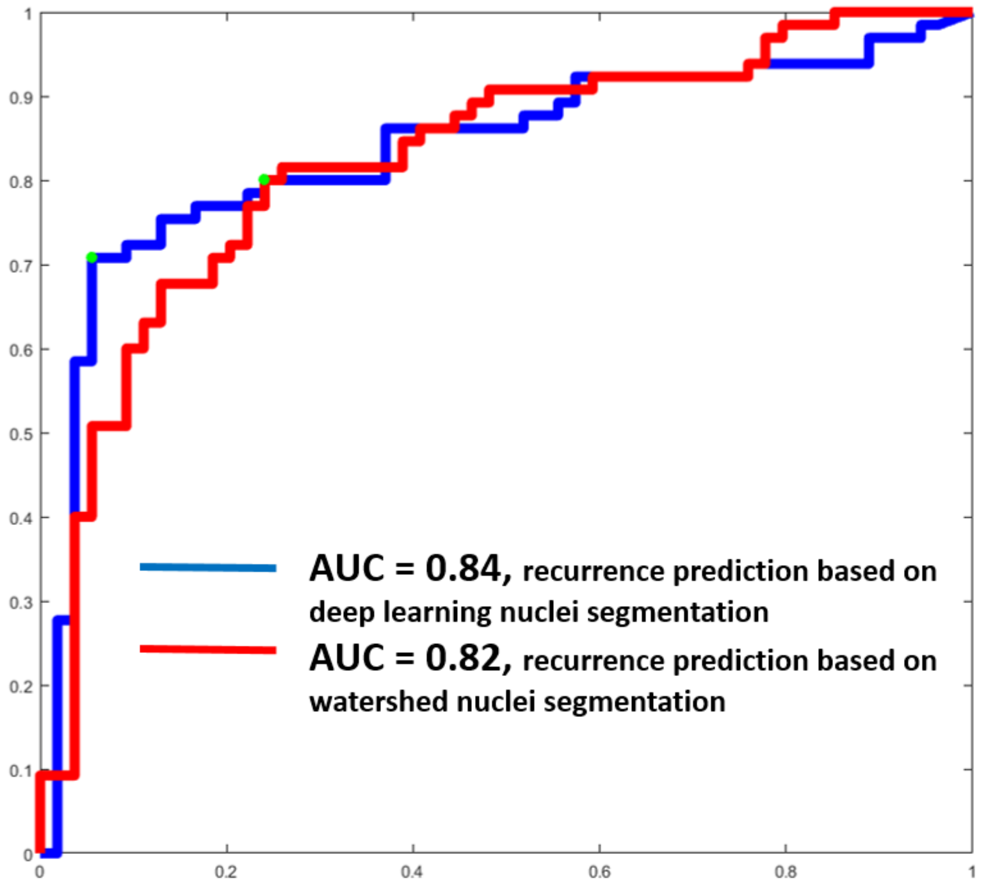

Figure 5. ROC analysis of recurrence prediction by our classifier on independent validation cohort #2 (n = 119). The prediction AUC based on watershed and deep learning nuclei segmentation are not statistically different (p-value = 0.2478), suggesting that our features are relatively robust to the nuclei segmentation approach used.

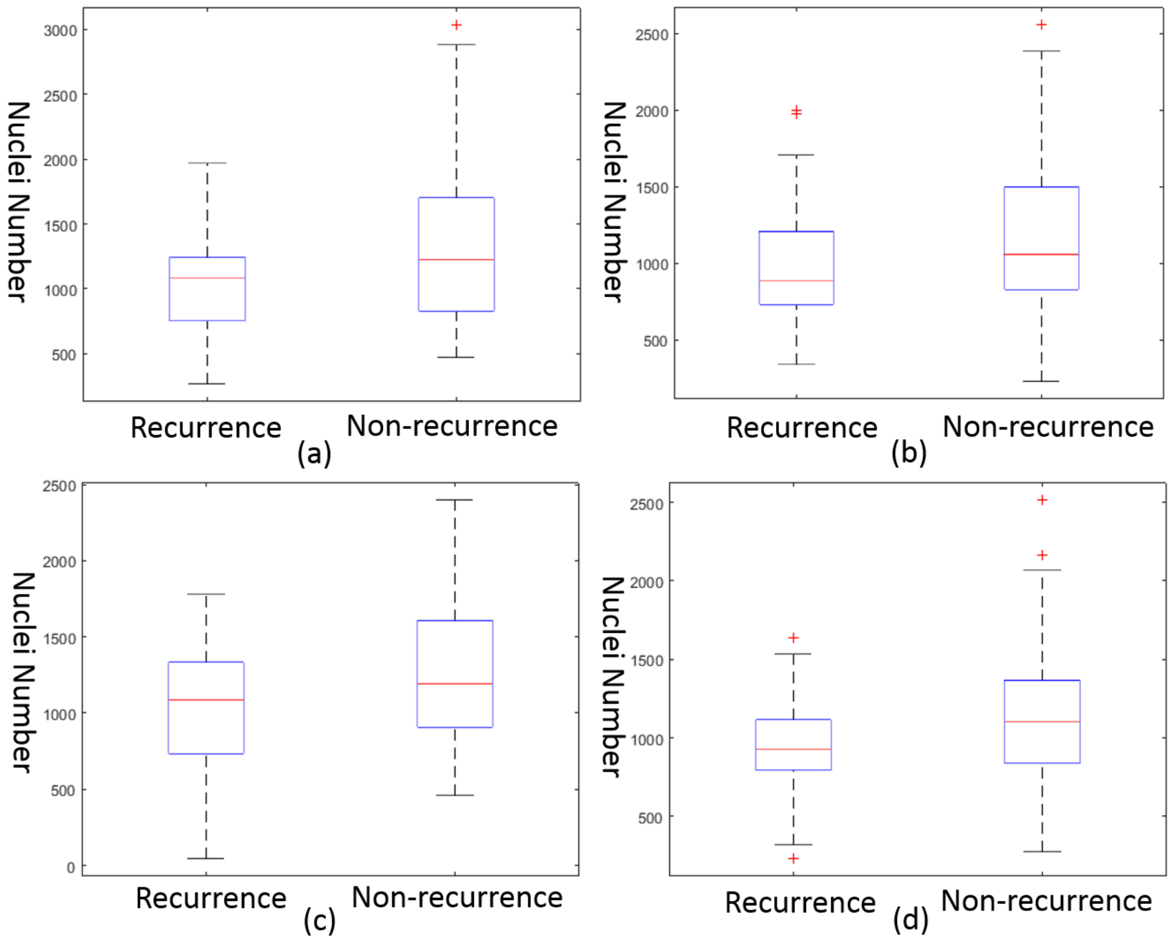

Figure 6. Boxplot of nuclei number for recurrence versus non-recurrence patients for (a) training cohort #1 by deep learning approach; (b) validation cohort #2 by deep learning approach; (c) training cohort #1 by watershed approach; (d) validation cohort #2 by watershed approach. The difference in the total number of nuclei identified by the two nuclei segmentation approaches is small.

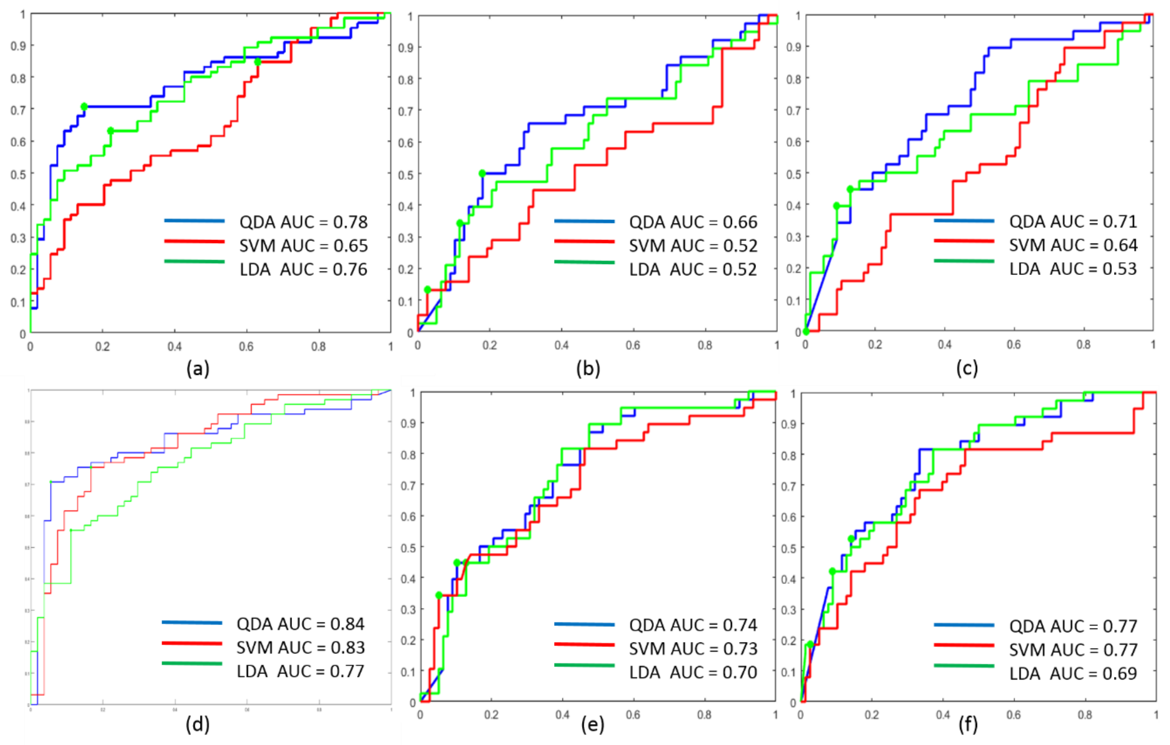

Figure 7. ROC analysis of classifiers prediction of recurrence built with nuclear shape and texture features only, as reported in 9, on validation (a) cohort #2 (n = 119), (b) cohort #3 batch #1 (n =116), and (c) cohort #3 batch #2 (n =116). Prediction AUC was lower and less consistent compared to our model which was built by including nuclear shape, texture and architecture features on validation (d) cohort #2 (n = 119), (e) cohort #3 batch #1 (n =116), and (f) cohort #3 batch #2 (n =116).

Table 1. Details of 242 feature descriptors

Global graph features	Shpae features	Orientation entropy	Local nuclear gluster graph	texture
'Voronoi:Area Standard Deviation'	'Morph:Mean Area Ratio'	OrientationEntropy:mean tensor contrast_energy'	local nuclear graph:Number of Nodes'	'Haralick:mean intensity contrast_energy'
'Voronoi:Area Average'	'Morph:Mean Distance Ratio'	OrientationEntropy:standard deviation tensor contrast_energy'	local nuclear graph:Number of Edges'	'Haralick:standard deviation intensity contrast_energy'
'Voronoi:Area Minimum / Maximum'	'Morph:Mean Standard Deviation of Distance'	OrientationEntropy:range tensor contrast_energy'	local nuclear graph:Average Degree'	'Haralick:mean intensity contrast_inverse_moment'
'Voronoi:Area Disorder'	'Morph:Mean Variance of Distance'	OrientationEntropy:mean tensor contrast_inverse_moment'	local nuclear graph:Average Eccentricity'	'Haralick:standard deviation intensity contrast_inverse_moment'
'Voronoi:Perimeter Standard Deviation'	'Morph:Mean Long/Short Distance Ratio'	OrientationEntropy:standard deviation tensor contrast_inverse_moment'	local nuclear graph:Diameter'	'Haralick:mean intensity contrast_ave'
'Voronoi:Perimeter Average'	'Morph:Mean Perimeter Ratio'	OrientationEntropy:range tensor contrast_inverse_moment'	local nuclear graph:Radius'	'Haralick:standard deviation intensity contrast_ave'
'Voronoi:Perimeter Minimum / Maximum'	'Morph:Mean Smoothness'	OrientationEntropy:mean tensor contrast_ave'	local nuclear graph:Average Eccentricity 90\%'	'Haralick:mean intensity contrast_var'
'Voronoi:Perimeter Disorder'	'Morph:Mean Invariant Moment 1'	OrientationEntropy:standard deviation tensor contrast_ave'	local nuclear graph:Diameter 90\%'	'Haralick:standard deviation intensity contrast_var'
'Voronoi:Chord Standard Deviation'	'Morph:Mean Invariant Moment 2'	OrientationEntropy:range tensor contrast_ave'	local nuclear graph:Radius 90\%'	'Haralick:mean intensity contrast_entropy'
'Voronoi:Chord Average'	'Morph:Mean Invariant Moment 3'	OrientationEntropy:mean tensor contrast_var'	local nuclear graph:Average Path Length'	'Haralick:standard deviation intensity contrast_entropy'
'Voronoi:Chord Minimum / Maximum'	'Morph:Mean Invariant Moment 4'	OrientationEntropy:standard deviation tensor contrast_var'	local nuclear graph:Clustering Coefficient C'	'Haralick:mean intensity intensity_ave'
'Voronoi:Chord Disorder'	'Morph:Mean Invariant Moment 5'	OrientationEntropy:range tensor contrast_var'	local nuclear graph:Clustering Coefficient D'	'Haralick:standard deviation intensity intensity_ave'
'Delaunay:Side Length Minimum / Maximum'	'Morph:Mean Invariant Moment 6'	OrientationEntropy:mean tensor contrast_entropy'	local nuclear graph:Clustering Coefficient E'	'Haralick:mean intensity intensity_variance'
'Delaunay:Side Length Standard Deviation'	'Morph:Mean Invariant Moment 7'	OrientationEntropy:standard deviation tensor contrast_entropy'	local nuclear graph:Number of connected components'	'Haralick:standard deviation intensity intensity_variance'
'Delaunay:Side Length Average'	'Morph:Mean Fractal Dimension'	OrientationEntropy:range tensor contrast_entropy'	local nuclear graph:giant connected component ratio'	'Haralick:mean intensity intensity_entropy'
'Delaunay:Side Length Disorder'	'Morph:Mean Fourier Descriptor 1'	OrientationEntropy:mean tensor intensity_ave'	local nuclear graph:average connected component size'	'Haralick:standard deviation intensity intensity_entropy'
'Delaunay:Triangle Area Minimum / Maximum'	'Morph:Mean Fourier Descriptor 2'	OrientationEntropy:standard deviation tensor intensity_ave'	local nuclear graph:number isolated nodes'	'Haralick:mean intensity entropy,'
'Delaunay:Triangle Area Standard Deviation'	'Morph:Mean Fourier Descriptor 3'	OrientationEntropy:range tensor intensity_ave'	local nuclear graph:percentage isolated nodes'	'Haralick:standard deviation intensity entropy,'
'Delaunay:Triangle Area Average'	'Morph:Mean Fourier Descriptor 4'	OrientationEntropy:mean tensor intensity_variance'	local nuclear graph:number end nodes'	'Haralick:mean intensity energy'
'Delaunay:Triangle Area Disorder'	'Morph:Mean Fourier Descriptor 5'	OrientationEntropy:standard deviation tensor intensity_variance'	local nuclear graph:percentage end nodes'	'Haralick:standard deviation intensity energy'

'MST:MST Edge Length Average'	'Morph:Mean Fourier Descriptor 6'	OrientationEntropy:range tensor intensity_variance'	local nuclear graph:number central nodes'	'Haralick:mean intensity correlation'
'MST:MST Edge Length Standard Deviation'	'Morph:Mean Fourier Descriptor 7'	OrientationEntropy:mean tensor intensity_entropy'	local nuclear graph:percentage central nodes'	'Haralick:standard deviation intensity correlation'
'MST:MST Edge Length Minimum / Maximum'	'Morph:Mean Fourier Descriptor 8'	OrientationEntropy:standard deviation tensor intensity_entropy'	local nuclear graph:mean edge length'	'Haralick:mean intensity information_measure1'
'MST:MST Edge Length Disorder'	'Morph:Mean Fourier Descriptor 9'	OrientationEntropy:range tensor intensity_entropy'	local nuclear graph:standard deviation edge length'	'Haralick:standard deviation intensity information_measure1'
'Arch:Area of polygons'	'Morph:Mean Fourier Descriptor 10'	OrientationEntropy:mean tensor entropy,'	local nuclear graph:skewness edge length'	'Haralick:mean intensity information_measure2'
'Arch:Number of Polygons'	'Morph:Standard Deviation Area Ratio'	OrientationEntropy:standard deviation tensor entropy,'	local nuclear graph:kurtosis edge length'	'Haralick:standard deviation intensity information_measure2'
'Arch:Density of Polygons'	'Morph:Standard Deviation Distance Ratio'	OrientationEntropy:range tensor entropy,'		
'Arch:Average distance to 3 Nearest Neighbors'	'Morph:Standard Deviation Standard Deviation of Distance'	OrientationEntropy:mean tensor energy'		
'Arch:Average distance to 5 Nearest Neighbors'	'Morph:Standard Deviation Variance of Distance'	OrientationEntropy:standard deviation tensor energy'		
'Arch:Average distance to 7 Nearest Neighbors'	'Morph:Standard Deviation Long/Short Distance Ratio'	OrientationEntropy:range tensor energy'		
'Arch:Standard Deviation distance to 3 Nearest Neighbors'	'Morph:Standard Deviation Perimeter Ratio'	OrientationEntropy:mean tensor correlation'		
'Arch:Standard Deviation distance to 5 Nearest Neighbors'	'Morph:Standard Deviation Smoothness'	OrientationEntropy:standard deviation tensor correlation'		
'Arch:Standard Deviation distance to 7 Nearest Neighbors'	'Morph:Standard Deviation Invariant Moment 1'	OrientationEntropy:range tensor correlation'		
'Arch:Disorder of distance to 3 Nearest Neighbors'	'Morph:Standard Deviation Invariant Moment 2'	OrientationEntropy:mean tensor information_measure1'		
'Arch:Disorder of distance to 5 Nearest Neighbors'	'Morph:Standard Deviation Invariant Moment 3'	OrientationEntropy:standard deviation tensor information_measure1'		
'Arch:Disorder of distance to 7 Nearest Neighbors'	'Morph:Standard Deviation Invariant Moment 4'	OrientationEntropy:range tensor information_measure1'		
'Arch:Avg. Nearest Neighbors in a 10 Pixel Radius'	'Morph:Standard Deviation Invariant Moment 5'	OrientationEntropy:mean tensor information_measure2'		
'Arch:Avg. Nearest Neighbors in a 20 Pixel Radius'	'Morph:Standard Deviation Invariant Moment 6'	OrientationEntropy:standard deviation tensor information_measure2'		
'Arch:Avg. Nearest Neighbors in a 30 Pixel Radius'	'Morph:Standard Deviation Invariant Moment 7'	OrientationEntropy:range tensor information_measure2'		
'Arch:Avg. Nearest Neighbors in a 40 Pixel Radius'	'Morph:Standard Deviation Fractal Dimension'			
'Arch:Avg. Nearest Neighbors in a 50 Pixel Radius'	'Morph:Standard Deviation Fourier Descriptor 1'			
'Arch:Standard Deviation Nearest Neighbors in a 10 Pixel Radius'	'Morph:Standard Deviation Fourier Descriptor 2'			
'Arch:Standard Deviation Nearest Neighbors in a 20 Pixel Radius'	'Morph:Standard Deviation Fourier Descriptor 3'			
'Arch:Standard Deviation Nearest Neighbors in a 30 Pixel Radius'	'Morph:Standard Deviation Fourier Descriptor 4'			
'Arch:Standard Deviation Nearest Neighbors in a 40 Pixel Radius'	'Morph:Standard Deviation Fourier Descriptor 5'			
'Arch:Standard Deviation Nearest Neighbors in a 50 Pixel Radius'	'Morph:Standard Deviation Fourier Descriptor 6'			
'Arch:Disorder of Nearest Neighbors in a 10 Pixel Radius'	'Morph:Standard Deviation Fourier Descriptor 7'			
'Arch:Disorder of Nearest Neighbors in a 20 Pixel Radius'	'Morph:Standard Deviation Fourier Descriptor 8'			
'Arch:Disorder of Nearest Neighbors in a 30 Pixel Radius'	'Morph:Standard Deviation Fourier Descriptor 9'			
'Arch:Disorder of Nearest Neighbors in a 40 Pixel Radius'	'Morph:Standard Deviation Fourier Descriptor 10'			
'Arch:Disorder of Nearest Neighbors in a 50 Pixel Radius'	'Morph:Median Area Ratio'			
	'Morph:Median Distance Ratio'			
	'Morph:Median Standard Deviation of Distance'			
	'Morph:Median Variance of Distance'			
	'Morph:Median Long/Short Distance Ratio'			
	'Morph:Median Perimeter Ratio'			
	'Morph:Median Smoothness'			
	'Morph:Median Invariant Moment 1'			
	'Morph:Median Invariant Moment 2'			
	'Morph:Median Invariant Moment 3'			
	'Morph:Median Invariant Moment 4'			
	'Morph:Median Invariant Moment 5'			
	'Morph:Median Invariant Moment 6'			
	'Morph:Median Invariant Moment 7'			
	'Morph:Median Fractal Dimension'			
	'Morph:Median Fourier Descriptor 1'			
	'Morph:Median Fourier Descriptor 2'			
	'Morph:Median Fourier Descriptor 3'			
	'Morph:Median Fourier Descriptor 4'			
	'Morph:Median Fourier Descriptor 5'			
	'Morph:Median Fourier Descriptor 6'			
	'Morph:Median Fourier Descriptor 7'			
	'Morph:Median Fourier Descriptor 8'			
	'Morph:Median Fourier Descriptor 9'			
	'Morph:Median Fourier Descriptor 10'			
	'Morph:Min / Max Area Ratio'			
	'Morph:Min / Max Distance Ratio'			
	'Morph:Min / Max Standard Deviation of Distance'			
	'Morph:Min / Max Variance of Distance'			
	'Morph:Min / Max Long/Short Distance Ratio'			
	'Morph:Min / Max Perimeter Ratio'			
	'Morph:Min / Max Smoothness'			
	'Morph:Min / Max Invariant Moment 1'			
	'Morph:Min / Max Invariant Moment 2'			
	'Morph:Min / Max Invariant Moment 3'			
	'Morph:Min / Max Invariant Moment 4'			

	'Morph:Min / Max Invariant Moment 5'			
	'Morph:Min / Max Invariant Moment 6'			
	'Morph:Min / Max Invariant Moment 7'			
	'Morph:Min / Max Fractal Dimension'			
	'Morph:Min / Max Fourier Descriptor 1'			
	'Morph:Min / Max Fourier Descriptor 2'			
	'Morph:Min / Max Fourier Descriptor 3'			
	'Morph:Min / Max Fourier Descriptor 4'			
	'Morph:Min / Max Fourier Descriptor 5'			
	'Morph:Min / Max Fourier Descriptor 6'			
	'Morph:Min / Max Fourier Descriptor 7'			
	'Morph:Min / Max Fourier Descriptor 8'			
	'Morph:Min / Max Fourier Descriptor 9'			
	'Morph:Min / Max Fourier Descriptor 10'			
